# Correlations between brain structures and study time at home in healthy children: a longitudinal analysis

**DOI:** 10.1002/brb3.278

**Published:** 2014-10-17

**Authors:** Michiko Asano, Yasuyuki Taki, Hiroshi Hashizume, Hikaru Takeuchi, Benjamin Thyreau, Yuko Sassa, Kohei Asano, Ryuta Kawashima

**Affiliations:** 1 Division of Developmental Cognitive Neuroscience, Institute of Development, Aging and Cancer, Tohoku UniversitySendai, Japan; 2 Department of Nuclear Medicine & Radiology, Institute of Development, Aging and Cancer, Tohoku UniversitySendai, Japan; 3 Division of Medical Neuroimaging Analysis, Department of Community Medical Supports, Tohoku Medical Megabank Organization, Tohoku UniversitySendai, Japan; 4 Smart Ageing International Research Centre, Institute of Development, Aging and Cancer, Tohoku UniversitySendai, Japan; 5 Department of Functional Brain Imaging, Institute of Development, Aging and Cancer, Tohoku UniversitySendai, Japan

**Keywords:** Children, study time at home, verbal, white matter volume

## Abstract

**Introduction:**

Like sleeping and eating habits, the study habits adopted by children when they are at home are important contributors to lifestyle and they affect cognitive ability. It has recently been reported that sleeping and eating habits change the brain structure of children. However, no research on the effect of study habits at home on the brain structure of children has been conducted thus far. We investigated the effects of study habits at home on the brain structures of healthy children by examining correlations between study time at home and changes in brain structure over the course of 3 years.

**Methods:**

We used the brain magnetic resonance images of 229 healthy children aged 5.6–18.4 years and computed the changes (time 2–time 1) in regional gray matter and white matter volume (rWMV) using voxel-based morphometry. Whole-brain multiple regression analysis revealed a significant positive correlation between study time at home and changes in rWMV in the right superior frontal gyrus (SFG). Behaviorally, we found a significant positive correlation between study time at home and change in the verbal comprehension index (VCI), one of the subscales of the Wechsler Intelligence Scale for Children–third edition (WISC–III).

**Results and Conclusions:**

Given that the SFG is involved in memory control and that the VCI measures abilities related to vocabulary, our results indicate that greater SFG involvement in the memorization component of longer study times may result in greater increases in the number of axons and more axon branching and myelination, causing plastic changes in the neural network involved in memory processes.

## Introduction

Lifestyle can have a positive or a negative influence on cognitive functioning and brain structure. A healthy lifestyle and intelligence have been associated with slower cognitive decline throughout life (Scarmeas and Stern 2003). In adults, engagement in intellectual and leisure-time social activities is associated with slowing or preventing declines in cognitive functions such as verbal intelligence (Hultsch et al. 1993; Gold et al. 1998). In contrast, certain lifestyles can negatively influence cognitive functioning and the brain. For example, it has been reported that type 2 diabetes, which is thought to be attributable primarily to lifestyle factors and genetics, causes a decline in cognitive functions such as declarative memory, which is associated with hippocampal atrophy (Awad et al. 2004). Obesity is among the conditions that are commonly associated with lifestyle. Taki et al. (2008) demonstrated that obesity is associated with decreased gray matter volume (GMV) and atrophy of the brain. Such obesity-related decreases in gray matter have also been observed in the prefrontal cortex (Pannacciulli et al. 2006) and medial temporal lobe (Gustafson et al. 2004). Alcohol consumption is one of the lifestyle-related factors that can influence cognitive functioning. Indeed, alcohol consumption disturbs the process by which memories are constructed in the hippocampus (Goodwin et al. 1969), and alcoholism is associated with memory disorders and functional depression via its effect on the prefrontal cortex (Kril and Halliday 1999; Moselhy et al. 2001).

The daily lifestyles of children influence the activities in which they engage and their academic achievement at school (Hofferth and Sandberg 2001). Children with a lifestyle that includes reading and studying at home tend to have better verbal ability (Snow et al. 1998) and academic achievement (Keith et al. 1986). Children who tend to watch TV for many hours tend to spend less time reading and studying (Koolstra and van der Voort 1996) and score lower on cognitive tests (Timmer et al. 1985). According to a survey of the Japanese public on the lifestyles and academic achievements of school children, insufficient sleep led to poor academic achievement (Hiroshima city 2003; Yamaguchi prefecture 2006). Not eating breakfast also adversely affected children's growth and academic achievement (MEXT 2006, 2007, 2008, 2009, 2010, 2011, 2012). As in adults, engaging in intellectual activity outside of school, such as studying at home, is thought to enhance children's cognitive development. In another Japanese survey, children who spent more time studying at home tended to have higher levels of academic achievement (Nemuro City, Japan 2011). Unlike the typical class work at school, studying at home primarily involves independent behavior. Working in the domain of educational psychology, Jean Piaget who was a developmental psychologist suggested that such individual independent activities promote children's cognitive development.

Only a few studies have examined the correlation between lifestyle and brain structure in healthy children. For example, Taki et al. (2010) examined the relationships between type of breakfast staple, brain GMV, and Intelligence Quotient (IQ) using brain magnetic resonance imaging (MRI) in healthy children and found that an appropriate breakfast was important for children's brain development. A significant positive correlation between sleep duration and GMV in the hippocampus, which is involved in memory, has also been reported in healthy children (Taki et al. 2012).

Many studies have been conducted regarding the correlation between learning or training and brain structure in adults. Extensive learning, such as medical students' preparation for their medical examinations, may result in increased GMV in the hippocampus and posterior parietal cortex (PPC; Draganski et al. 2006), and working-memory training may increase the fractional anisotropy of white matter in the intraparietal sulcus and anterior corpus callosum (Takeuchi et al. 2010b). Taken together, these results suggest that learning or training changes the brain structure in regions related to memory and improved cognitive ability.

Although children's study habits at home are among the important intellectual activities that influence their involvement in and academic achievement at school, no studies on the potential correlations between study habits at home and changes in brain structure have been performed to date. Furthermore, no research has clarified the specific cognitive ability that increases as a function of study time at home. More importantly, most relevant studies have been cross-sectional, which means they could not assess causality (i.e., does a certain lifestyle change brain structure, or does someone with a certain brain structure tend to have a certain lifestyle?).

Therefore, we investigated the effects of study habits at home on the brain structure of healthy children by examining the correlation between study time at home and changes in brain structure over the course of 3 years.

## Methods

### Participants

Right-handed, healthy Japanese children were recruited from kindergartens, elementary schools, junior high schools, and high schools in Miyagi Prefecture in Japan for participation in this study, which was conducted as a part of our research project on brain development in healthy children (Taki et al. 2011). A total of 302 children participated in the experiment at time 1, and 235 of these children participated in the experiment at time 2. The age range of participants was 5.6–18.4 years at time 1 (mean age, 12.20 years; standard deviation [SD], 3.08 years). All participants had normal vision, and none had a history of neurological or psychiatric illness. Handedness was evaluated using the Edinburgh Handedness Inventory (Oldfield 1971).

The function of the brain is different in the right-handed and the left-handed children. In the left-handed subjects, the probability of existence of the speech area is in the left hemisphere, which is different from that in the right-handed subjects (Pujol et al. 1999; Szaflarski et al. 2012). Previous studies, such as the cohort study of children using the MRI, mostly selected right-handed subjects (Berquin et al. 1998; Vance et al. 2007). Thus, our experiment also employed right-handed subjects. In accordance with the Declaration of Helsinki (World Medical Association 1991), written informed consent was obtained from each participant and his or her parent. Our study was approved by the Institutional Review Board of the Tohoku University Graduate School of Medicine.

A total of 234 subjects who joined our experiment at time 1 or time 2 were selected for MRI scanning. We excluded five subjects from these analyses: three were able to complete the psychological tests but were unable to remain in the MRI scanner during the scan, one had a developmental disability, and one was diagnosed with encephalic angioma based on the MR image. In addition, one subject preparing for a high school entrance examination at time 1 was also excluded because it was thought that this individual's study behavior at home at that time did not appropriately represent that individual's normal study habits. Ultimately, data from 229 subjects were analyzed (111 boys, 118 girls).

### IQ test

IQ was measured with the Japanese version of the age-appropriate Wechsler test, which was administered the same day as the MRI scans at times 1 and 2. The Wechsler Intelligence Scale for Children–third edition (WISC–III) was used for subjects younger than 16 years of age, and the Wechsler Adult Intelligence Scale–third edition (WAIS–III) was used for subjects older than 16.0 years of age (Wechsler 1991, 1997a,b; Wechsler and Psychological Corporation 1991). We also used the Verbal IQ (VIQ) and Performance IQ (PIQ) subscales of the Full-scale IQ for the analyses. The VIQ consists of the Verbal Comprehension Index (VCI) and the Freedom from Distractibility Index (FDI)/Working Memory Index (WMI); the PIQ consists of the Perceptual Organization Index (POI) and the Processing Speed Index (PSI). Our study focused on the subscales of the IQ test to identify the cognitive ability that is, associated with children's study habits at home.

### Study habits at home

At time 1, we used the following question to gather data about the time participants devoted to studying at home: “How long do you study at home on weekdays? Respondents answered by endorsing one of the following options: 1, never; 2, almost never; 3, <30 min; 4, about 30 min; 5, about 45 min; 6, about 1 h; 7, about 1 h 30 min; 8, about 2 h; 9, about 2 h 30 min; 10, about 3 h; 11, more than 3 h; 12, not sure. These choices were transformed as follows for the statistical analyses described below: 1 = 0 min, 2 = 10 min, 3 = 20 min, 4 = 30 min, 5 = 45 min, 6 = 60 min, 7 = 90 min, 8 = 120 min, 9 = 150 min, 10 = 180 min, 11 = more than 180. Data from a subject who chose 12 (not sure) were not used in the analyses. This questionnaire was filled by parents for children in fourth grade or less, and the subjects in fifth grade or higher filled it themselves. This was because of low reliability of answers from small children. Moreover, this drawing of a line between the fourth and fifth graders is according to standard practice in the field and previous recommendation (Kambara et al. 1998).

### Socioeconomic status

Given the significant correlation between annual family income and children's study time at home (Bianchi and Robinson 1997), we controlled for the effect of socioeconomic status. We collected data on annual family income information from subjects' parent(s) and treated the data as a discrete variable: 1, less than US$20,000 (the currency exchange rate was set at US$1 = 100 yen); 2, US$20,000 <40,000; 3, US$40,000 < 60,000; 4, US$60,000 < 80,000; 5, US$80,000 < 100,000; 6, US$100,000 < 120,000; 7, US$120,000 or more (Taki et al. 2010).

### Parents' educational background

As previous research has shown that children's study time at home is related to the educational background of their parents (Bianchi and Robinson 1997), we controlled for the effect of this variable. We asked subjects' parents to select the category that best described their educational background: 1, elementary school graduate or below; 2, junior high school graduate; 3, normal high school graduate; 4, graduate of a short-term school completed after high school (such as a junior college); 5, university graduate; 6, Masters degree; and 7, Doctorate. The average of the answers provided by the parents was used in the analyses.

### Image acquisition

All images were acquired with a 3-T MRI scanner (Philips Achieva). Three-dimensional, high-resolution, T1-weighted images (T1WI) were collected using a magnetization-prepared rapid gradient-echo (MPRAGE) sequence. The parameters were as follows: 240 × 240 matrix, TR = 6.5 ms, TE = 3 ms, TI = 711 ms, FOV = 24 cm, 162 slices, 1.0 mm slice thickness, and scan duration of 8 min and 3 s.

### Preprocessing

A series of preprocessing procedures was performed using Statistical Parametric Mapping 8 software (SPM8; Wellcome Department of Cognitive Neurology, London, UK) and Matlab (Mathworks, Natick, MA). First, using the new segmentation algorithm implemented in SPM8, T1-weighted structural images of each individual were segmented into gray matter tissue, white matter tissue, and cerebrospinal fluid (Ashburner and Friston 2005). Second, segmented GM/WM tissues for all subjects were used to create a customized template using Diffeomorphic Anatomical Registration Through Exponentiated Lie algebra (DARTEL; Ashburner 2007). DARTEL estimates a best set of smooth deformations from every subject's tissue to their common average and reiterates the process until convergence. In particular, we created the well-demarcated average image from subjects' segmented images, then fit subjects' segmented images to the average image, and reiterated those calculations. The resultant images were spatially normalized to MNI space with affine transformation to create the DARTEL template. Then, each subject's segmented images were normalized to the DARTEL template via nonlinear transformation. In the normalization process, we performed a volume change correction (modulation) by modulating each voxel with the Jacobian determinants derived from spatial normalization, allowing for the determination of regional differences in the absolute amount of brain tissue (Ashburner and Friston 2000). Subsequently, all of the images of regional gray and white matter volume (rGMV and rWMV, respectively) were smoothed by convolving them with an isotropic Gaussian kernel of 8 mm full-width at half-maximum (FWHM).

### Behavioral data analysis

The behavioral data were analyzed using the Statistical Package for the Social Sciences 17.0 (SPSS Inc, Chicago, IL). Multiple regression analyses were used to investigate the correlation between study time at home and changes in IQ subscales (VCI, POI, FDI/WMI, and PSI) between time 1 and time 2. Change in each subscale was treated as a dependent variable, and study time at home was treated as an independent variable. Age at time 1, duration, sex, annual family income, educational background of parents, and subscale scores at time 1 were also used as independent variables for the adjustment. The statistical threshold was set at *P *<* *0.05 for these behavioral analyses.

### Statistical analyses

Statistical analyses of imaging data were performed using VBM8 in SPM8. For the longitudinal analysis, the change in rGMV/rWMV between time 2 and time 1 (time 2–time 1) was computed at each voxel for each participant using ImCalc implemented in SPM8. In this computation, we included only voxels that showed GMV values >0.10 both at time 1 and at time 2 to effectively limit the images to areas likely to be GM/WM (Takeuchi et al. 2010a,b).

Whole-brain multiple regression analyses were performed to investigate the correlation between rGMV/rWMV and study time at home. Change in rGMV/rWMV was treated as a dependent variable, and study time at home was treated as an independent variable. Age at time 1, duration, sex, annual family income, educational background of parents, and intracranial volume at time 1 were also treated as independent variables for the adjustment. Statistical significance for these image analyses was set at *P *<* *0.05, family-wise error (FWE) corrected.

To check for any nonlinear effects of study time at home on changes in brain structure, we divided the subjects into three groups: short-time group (1, never; 2, almost never; 3, <30 min; 4, about 30 min), middle-time group (5, about 45 min; 6, about 1 h), and long-time group (7, about 1 h 30 min; 8, about 2 h; 9, about 2 h 30 min; 10, about 3 h) for multiple regression analyses by group.

### The post hoc investigation on the association between changes in rGMV/rWMV and VCI

To investigate whether VCI and anatomical correlates of the study time at home were associated in a post hoc manner, we applied multiple regression analysis to confirm the correlation between change in rGMV/rWMV and change in VCI, adjusting for age at time 1, duration, sex, annual family income, educational background of their parents, and Intracranial volume at time 1. Since we could not get any significant results in the whole-brain analysis, we conducted region of interest (ROI) analysis using small volume correction (SVC) in SPM8, where we set the ROI as a 5 mm radius sphere around the peak of the cluster based on the results of the correlation analysis. Statistical significance for these image analyses was set at *P *<* *0.05, family-wise error (FWE) corrected.

### Interactive effects between sex and study time at home on changes in rGMV/rWMV

In the whole-brain analysis, we used voxel-wise analysis of covariance (ANCOVA), with sex difference as a grouping factor. In this analysis, we used age at time 1, duration, sex, annual family income, educational background of their parents, and ICV at time 1 as covariates. Age at time 1, duration, sex, annual family income, educational background of their parents, ICV at time 1, and study time at home were modeled so that each covariate had a unique relationship with rGMV/rWMV for each sex (using the interactions option in SPM8), which established the investigation of the effects of interaction between sex and each covariate. The interactive effects between sex and study time at home on changes in rGMV/rWMV were applied using f-contrast. Statistical significance for these image analyses was set at *P *<* *0.05, family-wise error (FWE) corrected.

## Results

### Behavioral data

The characteristics of the subjects are presented in Tables 1–4. We found a significant positive correlation between study time at home and change in the VCI (*P *=0.038, *b *=* *0.134; Table 5) but no significant correlation between study time at home and changes in the other subscales (FDI/WMI, POI, and PSI). The group analysis revealed no significant correlations between study time at home and changes in any of the subscales in any group (Table 6).

**Table 1:** Intelligence Quotient (IQ) of the subjects at time 1

	All subjects (*n *=* *229)		Boys (*n *=* *111)		Girls (*n *=* *118)		*P*
Mean	SD	Mean	SD	Mean	SD		
FIQ	103.23	11.85	102.04	10.91	104.35	12.62	0.16
VIQ	104.31	12.64	102.76	13.04	105.77	12.13	0.31
PIQ	101.23	12.61	100.57	10.90	101.85	14.53	0.04*
VCI	104.24	13.78	103.20	14.06	105.23	13.41	0.40
POI	101.73	13.45	100.41	11.19	102.97	15.23	0.01*
FDI/WMI	100.24	12.66	98.80	11.97	101.58	13.19	0.28
PSI	101.89	12.91	103.75	13.13	100.14	12.58	0.75

FIQ, Full-scale IQ; VIQ, Verbal IQ; PIQ, Performance IQ; VCI, Verbal Comprehension Index; POI, Perceptual Organization Index; FDI, Freedom from Distractibility Index; WMI, Working Memory Index; PSI, Processing Speed Index.

* *P *<* *0.05.

**Table 2:** IQ of the subjects at time 2

	All subjects (*n *=* *229)		Boys (*n *=* *111)		Girls (*n *=* *118)		*P*
Mean	SD	Mean	SD	Mean	SD		
FIQ	104.76	12.20	104.28	12.07	105.22	12.36	0.86
VIQ	105.12	13.01	104.41	13.26	105.79	12.80	0.71
PIQ	103.40	12.97	103.32	12.01	103.47	13.87	0.18
VCI	105.90	13.71	105.50	13.54	106.29	13.91	0.93
POI	102.21	13.93	101.09	12.43	103.26	15.19	0.07*
FDI/WMI	99.31	13.00	97.84	12.11	100.70	13.69	0.44
PSI	107.19	12.78	109.03	12.78	105.46	12.59	0.73

FIQ, Full scale IQ; VIQ, Verbal IQ; PIQ, Performance IQ; VCI, Verbal Comprehension Index; POI, Perceptual Organization Index; FDI, Freedom from Distractibility Index; WMI, Working Memory Index; PSI, Processing Speed Index.

* *P *<* *0.05.

**Table 3:** Characteristics of the subjects

	All subjects (*n *=* *229)		Boys (*n *=* *111)		Girls (*n *=* *118)		*P*
Mean	SD	Mean	SD	Mean	SD		
Age at time 1	11.20	3.08	11.39	3.31	11.04	2.84	0.04*
Age at time 2	14.25	3.11	14.44	3.35	14.07	2.87	0.06
Study time at home	58.89	41.81	60.00	43.50	57.84	40.32	0.77
Family annual income	3.88	1.47	3.77	1.48	3.97	1.51	0.61
Educational background of parents	4.13	0.79	4.05	0.71	4.21	2.84	0.01*

* *P *<* *0.05.

**Table 4:** Three-year change in IQ and subscale scores (time 2–time 1)

	All subjects (*n *=* *229)		Boys (*n *=* *111)		Girls (*n *=* *118)		*P*
Mean	SD	Mean	SD	Mean	SD		
FIQ	1.54	8.67	2.24	8.20	0.87	9.07	0.37
VIQ	0.81	10.00	1.66	9.43	0.02	10.50	0.18
PIQ	2.17	9.86	2.76	9.68	1.62	10.04	0.58
VCI	1.66	11.11	2.30	10.48	1.06	11.69	0.14
POI	0.48	10.94	0.68	10.40	0.29	11.47	0.40
FDI/WMI	−0.92	9.66	−0.96	9.91	−0.88	9.47	0.37
PSI	5.30	11.23	5.28	11.93	5.31	10.57	0.17

**Table 5:** Partial regression coefficients in multiple regression analysis between changes in VCI, POI, FDI/WMI, and PSI and study time at home in all subjects

Subscale	Partial regression coefficient	*P*
VCI	0.134*	0.038
POI	0.012	0.863
FDI/WMI	−0.052	0.432
PSI	−0.012	0.844

* *P *<* *0.05.

**Table 6:** Partial regression coefficients in multiple regression analysis between changes in VCI and study time in short-time, middle-time, and long-time groups

	Partial regression coefficient	*P*
Short time	−0.101	0.284
Middle time	0.022	0.860
Long time	0.46	0.776

### Imaging data

**Figure 1:**
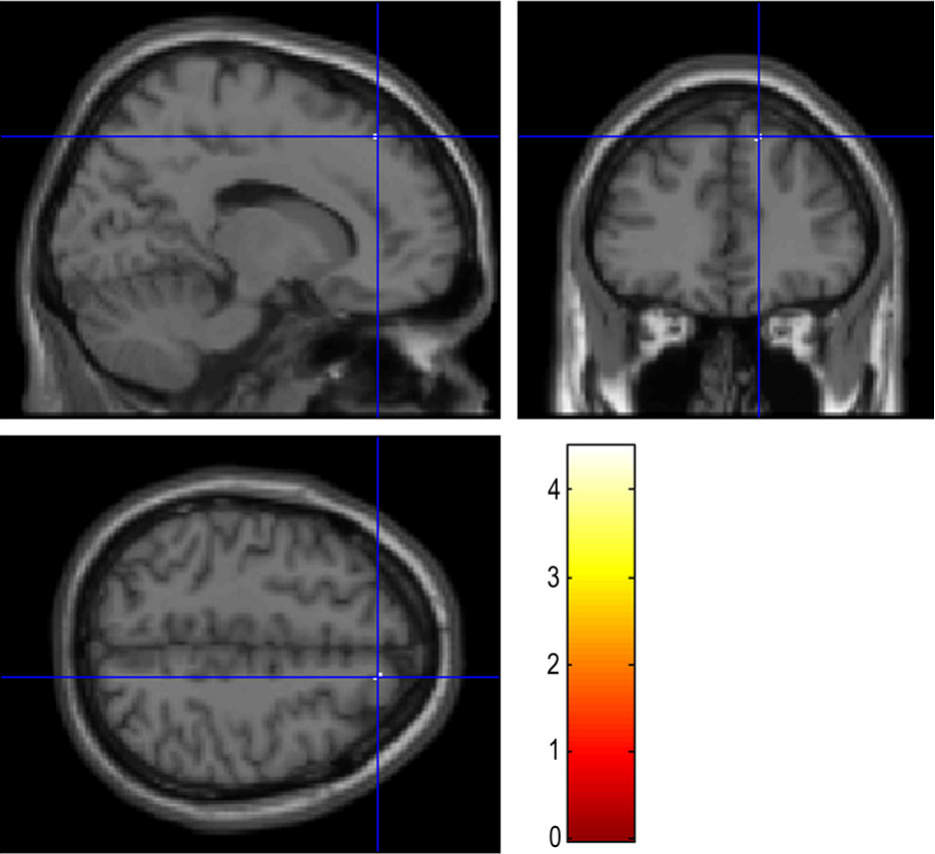
White matter region showing a significant positive correlation between 3-year change in rWMV and study time at home in all subjects. *P *<* *0.05 family-wise error (FWE) corrected for multiple comparisons. The region with a significant correlation is the white matter region adjacent to the right superior frontal gyrus.

**Figure 2:**
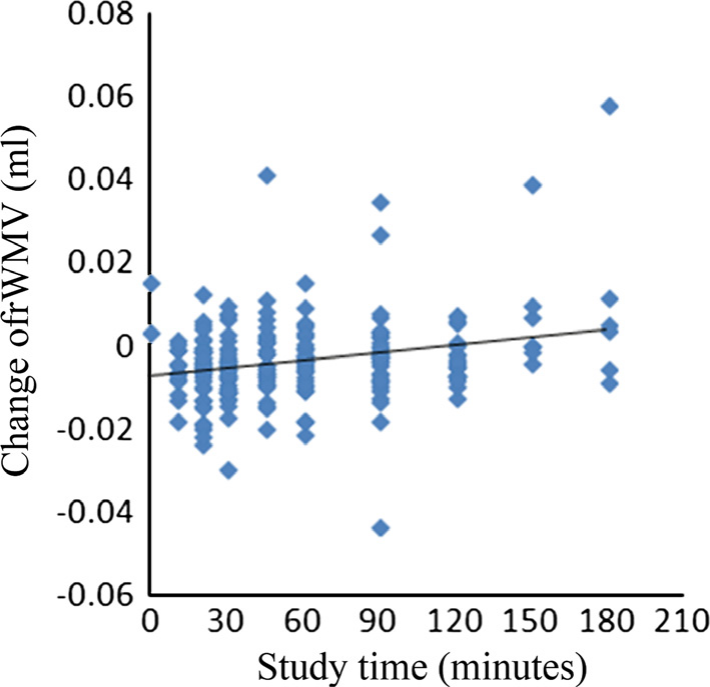
Scatter plot of relationship between changes in rWMV in the right superior frontal gyrus and study time at home in all subjects. A line fitted by linear regression is also shown.

The whole-brain analyses revealed a significant positive correlation between study time at home and rWMV in the right superior frontal gyrus (SFG), *P *<* *0.05 FWE corrected (Figs.1, 2, Table 7). No brain regions showed a statistically significant correlation between study time at home and rGMV.

**Table 7:** Brain areas showing statistically significant correlation between change in rWMV and study time at home (*P *<* *0.05, family-wise error (FWE) corrected for multiple comparisons)

	Correlation	Size	Brain region	*x*	*y*	*z*	*t*
All subjects	Positive	4	Right superior frontal gyrus	15	38	49	4.49
Long time	Positive	154	Right precuneus	21	−48	52	5.51
			Right precuneus	17	−48	42	5.08
			Right precuneus	27	−42	40	4.99
	Positive	3	Superior temporal gyrus	−44	−46	7	4.55

**Figure 3:**
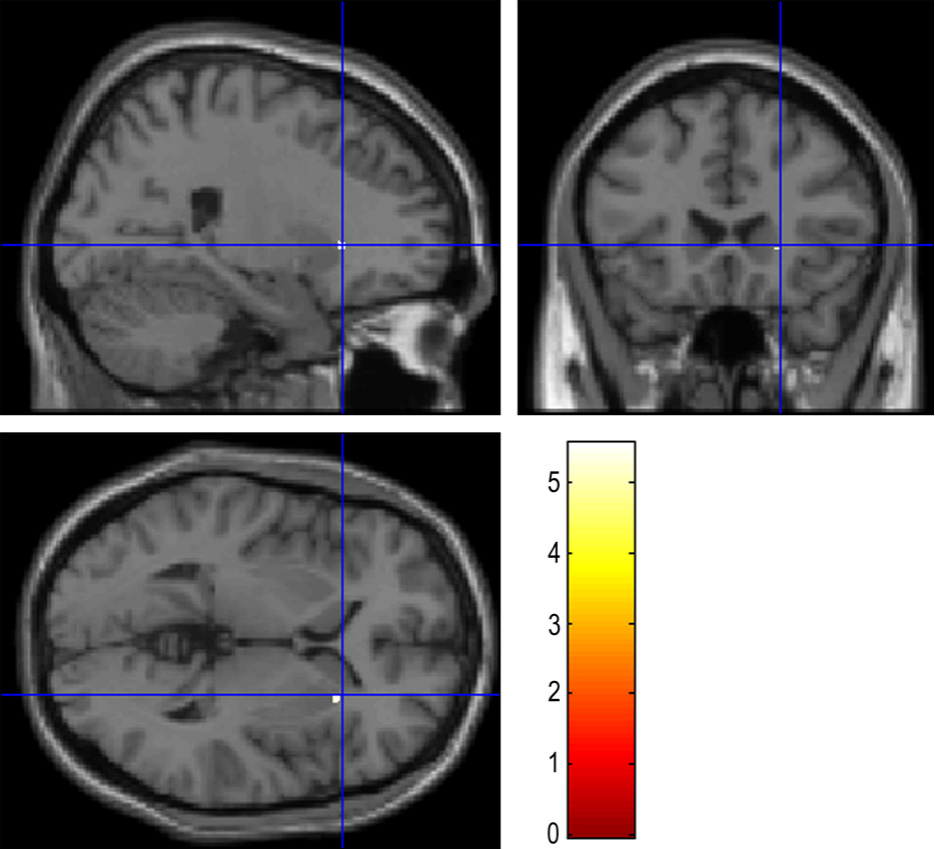
White matter region showing a significant negative correlation between 3-year change in the rWMV and study time at home in the middle-time group. *P *<* *0.05 family-wise error (FWE) corrected for multiple comparisons. The region with a significant negative correlation is shown in the white matter region adjacent to the right caudate nucleus.

**Figure 4:**
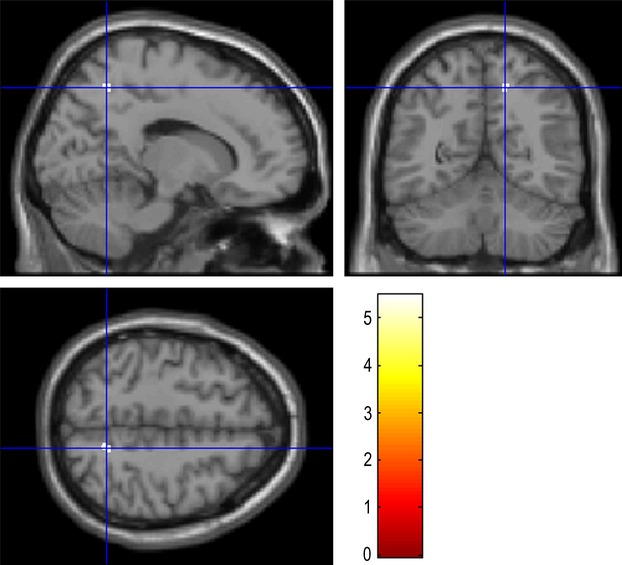
Gray matter region with a significant positive correlation between 3-year change in rGMV and study time at home in long-time group. *P *<* *0.05 family-wise error (FWE) corrected for multiple comparisons. The region with a significant positive correlation is shown in the right precuneus.

**Figure 5:**
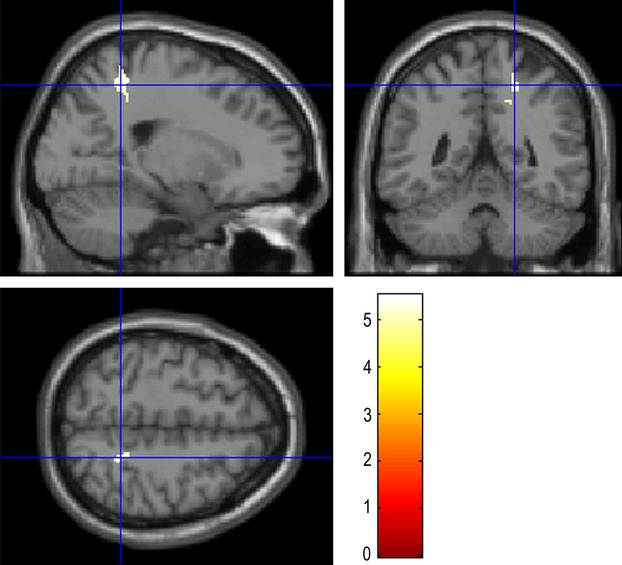
White matter region showing a significant positive correlation between 3-year changes in rWMV and study time at home in long-time group. *P *<* *0.05 family-wise error (FWE) corrected for multiple comparisons. The region with a significant positive correlation is shown in the white matter region adjacent to the right precuneus.

The group analysis revealed no statistically significant correlations between study time at home and rWMV/rGMV in the short-time group. However, we found a significant negative correlation between study time at home and rWMV in the caudate nucleus in the middle-time group, *P *<* *0.05, FWE corrected (Fig.3, Table 8). We also found a significant positive correlation between study time at home and change in rGMV in the right precuneus and the right inferior parietal lobe in the long-time group, *P *<* *0.05, FWE corrected (Fig.4, Table 8). A significant positive correlation between study time at home and change in rWMV was found in the right precuneus and the right superior temporal gyrus in the long-time group, *P *<* *0.05 FWE corrected (Fig.5, Table 7).

**Table 8:** Areas showing statistically significant correlation between change in rGMV and study time at home (*P *<* *0.05, family-wise error (FWE) corrected for multiple comparisons)

	Correlation	Size	Brain region	*x*	*y*	*z*	*t*
Middle time	Negative	12	Right caudate nucleus	24	23	1	5.55
Long time	Positive	27	Right precuneus	15	−57	51	5.48
	Positive	20	Right inferior parietal lobule	48	−36	46	5.39
	Positive	20	Right supramarginal gyrus	57	−16	24	5.23

### The post hoc investigation on the association between changes in rGMV/rWMV and VCI

There was a significant positive correlation between changes in VCI score and rWMV in the right SFG in the ROI analysis (*x *=* *15, *y *=* *41, *z *=* *46, *P* < 0.05; FWE corrected within the ROI). The results suggest an association between observed anatomical changes caused by study time at home and VCI changes.

### Interactive effects between sex and study time at home on changes in rGMV/rWMV

There were no significant effects of interactions between sex and the study time at home observed on rGMV/rWMV.

## Discussion

This is the first study to investigate the relationship between study time at home and structural brain changes in healthy children using a longitudinal design. A statistically significant positive correlation between study time at home and rWMV was observed in the SFG.

The whole-brain analyses revealed a significant positive correlation between study time at home and rWMV in the right SFG. This means that longer study time was associated with an increase in rWMV in the SFG. The prefrontal cortex, including the SFG, is known to play an important role in memory control. Increased rWMV has been thought to be associated with changes in the number of axons, axon diameter, axon branching, axon trajectory, and myelination (Zatorre et al. 2012). Neural fibers in the PFC, including the SFG, project primarily to the parietal lobe (Makris et al. 2007; Schmahmann and Pandya 2007) and hippocampus (Goldmanrakic et al. 1984; Laroche et al. 2000). In particular, the connection between the PFC and the hippocampus is thought to form a neural network that is, involved in learning and memory (Damasio 1989; Squire 1992). Memory formation involves not only certain brain regions (Gold et al. 1998; Eichenbaum 2002), but also reciprocal connections between the frontal lobe and the hippocampus (Wall and Messier 2001; Shastri 2002). For example, the PFC plays an important role in the reorganization and retrieval of long-term implicit memories (Shastri 2002; Ranganath et al. 2003). In addition, cognitively based learning and training affect brain structure. For example, it was found that learning to juggle increased the rWMV in the PPC (Draganski et al. 2004). In addition, working-memory training has also been found to strengthen the fractional anisotropy of WM in the intraparietal sulcus and the anterior corpus callosum (Takeuchi et al. 2010a). Taken together, our results suggest that greater recruitment of the SFG in the memorization process during longer study times may result in greater increases in the number of axons as well as more axon branching and myelination, thereby causing plastic changes in the neural network involved in memory processing.

The group analysis revealed different correlations between study time at home and changes in brain structure according to the brain structure under analysis, implying that study time at home had a nonlinear effect on brain structure or that a more complicated mechanism, such as region dependency, was involved. In the long-time group, we found a significant positive correlation between study time at home and change in rGMV and rWMV in the right precuneus. The precuneus is reportedly involved in the retrieval of episodic memory, which is a part of declarative memory (Tulving et al. 1996). The precuneus is activated during the retrieval of memories of personal experiences (Buckner et al. 1996; Tulving et al. 1996; Henson et al. 1999). Taken together, these data indicate that study habits at home and, more specifically, the retrieval of memories, may affect the structure of the precuneus. Furthermore, study time at home may have nonlinear effects on changes in brain structures; that is, studying may significantly affect brain structures only when enough time has been devoted to this activity (e.g., more than 90 min).

In the middle-time group, study time at home was significantly negatively correlated with rGMV in the caudate nucleus. Although it is difficult to interpret this correlation, the recruitment of this structure may be related to the control of cognitive information and working memory during studying at home. Indeed, the caudate nucleus is one of the regions in the prefrontal loop, which is involved in attention and decision-making via the control of cognitive information and working memory (Alexander et al. 1986; Middleton and Strick 2000). Previous studies have shown a significant negative correlation between GM thickness and cognitive ability (Lu et al. 2007; Takeuchi et al. 2010b), implying that more synaptic pruning and myelination is associated with greater cognitive ability (Takeuchi et al. 2010b). Taken together, our results suggest that synaptic pruning and myelination may occur in the caudate nucleus through the recruitment of working memory and decision-making operations during studying at home.

The behavioral analysis revealed a significant positive correlation between study time at home and 3-year changes in VCI scores. Given that the VCI measures verbal ability with regard to words and knowledge, this may imply that longer study time at home increased this ability. Previous studies have found that study time at home plays an important role in enhancing academic achievement (Hofferth and Sandberg 2001; Nemuro City, Japan 2011), supporting our results. Studies showing that study habits at home enhance verbal ability (Keith et al. 1986; Snow et al. 1998) also support our results. Moreover, given that more independence is involved in studying at home than in learning at school and that such independent intellectual activity increases cognitive ability (Piaget 1964), it is plausible that a longer study time at home may increase cognitive ability by virtue of its reliance on more independent intellectual activity.

As the behavioral group analysis did not reveal a statistically significant correlation between study time at home and changes in the VCI, our results do not support the possibility of a nonlinear effect of study time at home on the VCI. The present study has some limitations. First, study time at home was measured only at time 1. Given that children's lifestyles gradually change as they advance in school (Hofferth and Sandberg 2001), their study habits at home may have changed in the 3 years following time 1. However, in the longitudinal analysis for the purpose of this study, the only essential variable is the study time at time 1 because this project is a prospective longitudinal cohort study. Moreover, to indicate the causality at a certain level in these kinds of prospective longitudinal cohort study analyses, it is important that certain variables at time 1 predict the subsequent changes in the other variables. Otherwise, the analyses cannot suggest stronger causality than the cross-sectional studies.

A cross-sectional survey of the public about the lifestyles of children in Japan reported that fifth-grade students in elementary school and second-year students in junior high school had comparable study times at home (Cabin office 2001), supporting the validity of our analysis. Future research should examine lifestyle changes as a function of development. Second, in the multiple comparison analyses of imaging data, we assumed a linear effect of developmental changes on rGMV/rWMV; that is, we assumed an age effect and controlled for it. However, the developmental trajectory of GM takes the shape of an inverted-U curve with a peak at 9–10 years of age (Lenroot and Giedd 2006). Such a nonlinear effect was not considered in this study.

In conclusion, we found that children's study habits at home influenced not only their cognitive ability but also the structure of their brains at some point during the course of 3 years. Our results may help clarify the mechanism underlying the effects of lifestyle on the brains of children.
